# Associations between mild-to-moderate anaemia in pregnancy and helminth, malaria and HIV infection in Entebbe, Uganda

**DOI:** 10.1016/j.trstmh.2007.03.017

**Published:** 2007-09

**Authors:** Lawrence Muhangi, Patrick Woodburn, Mildred Omara, Nicholas Omoding, Dennison Kizito, Harriet Mpairwe, Juliet Nabulime, Christine Ameke, Linda A. Morison, Alison M. Elliott

**Affiliations:** a MRC/UVRI Uganda Research Unit on AIDS, Uganda Virus Research Institute, P.O. Box 49, Entebbe, Uganda; b Entebbe Hospital, P.O. Box 29, Entebbe, Uganda; c London School of Hygiene & Tropical Medicine, Keppel Street, London WC1E 7HT, UK

**Keywords:** Anaemia, Helminth, Pregnancy, HIV, Malaria, Uganda

## Abstract

It is suggested that helminths, particularly hookworm and schistosomiasis, may be important causes of anaemia in pregnancy. We assessed the associations between mild-to-moderate anaemia (haemoglobin >8.0 g/dl and <11.2 g/dl) and helminths, malaria and HIV among 2507 otherwise healthy pregnant women at enrolment to a trial of deworming in pregnancy in Entebbe, Uganda. The prevalence of anaemia was 39.7%. The prevalence of hookworm was 44.5%, *Mansonella perstans* 21.3%, *Schistosoma mansoni* 18.3%, *Strongyloides* 12.3%, *Trichuris* 9.1%, *Ascari*s 2.3%, asymptomatic *Plasmodium falciparum* parasitaemia 10.9% and HIV 11.9%. Anaemia showed little association with the presence of any helminth, but showed a strong association with malaria (adjusted odds ratio (AOR) 3.22, 95% CI 2.43–4.26) and HIV (AOR 2.46, 95% CI 1.90–3.19). There was a weak association between anaemia and increasing hookworm infection intensity. Thus, although highly prevalent, helminths showed little association with mild-to-moderate anaemia in this population, but HIV and malaria both showed a strong association. This result may relate to relatively good nutrition and low helminth infection intensity. These findings are pertinent to estimating the disease burden of helminths and other infections in pregnancy. [Clinical Trial No. ISRCTN32849447]

## 1 Introduction

Anaemia in pregnancy contributes to maternal deaths and may also contribute to adverse birth outcomes including intrauterine growth retardation and prematurity, and hence to perinatal morbidity and mortality (WHO, 1991).

Hookworm is an important cause of anaemia in developing countries, but lack of consensus regarding the risks and benefits of treating helminths in pregnancy has, until recently, led to a tendency to exclude pregnant and even breastfeeding women from deworming programmes. In 1994, a call was made for research leading to improved estimates of hookworm infection in women of child-bearing age and for evaluation of interventions that might be beneficial (WHO, 1994) but, to our knowledge, only one placebo-controlled trial of the treatment of hookworm in pregnancy has been reported (Torlesse and Hodges, 2000, Torlesse and Hodges, 2001).

More recently, an Informal Consultation held by the WHO gave consideration to the possible effects of schistosomiasis in pregnancy and it was suggested that anaemia might be among them (Allen et al., 2002, WHO, 2002). Although praziquantel had been widely avoided in pregnant and lactating women, it was noted that there was no evidence from studies in animals, from case reports or from mass treatment campaigns in humans of any major adverse effects. Treatment of schistosomiasis during pregnancy was therefore advocated by the Consultation Committee.

We have undertaken a trial designed to examine the effects of maternal helminths and of deworming during pregnancy on the response to immunisation and susceptibility to infection and disease in infancy [ISRCTN32849447] (Elliott et al., 2007). Anaemia is among the secondary outcomes of the trial. The trial is ongoing. In view of current interest in the role of helminths in anaemia during pregnancy, we have examined associations between anaemia and helminths and other major infections (malaria and HIV) among pregnant women at enrolment into the trial.

## 2 Materials and methods

### 2.1 Study population and procedures

The study area comprises Entebbe Municipality and the adjacent subcounty of Katabi. This area supports semi-urban, rural and fishing communities residing on the Entebbe peninsula in Lake Victoria, Uganda. Women were recruited at the antenatal clinic at Entebbe Hospital between April 2003 and November 2005.

Women were assessed for screening at their first antenatal visit, thus initial screening could take place in any trimester of pregnancy. They were eligible for screening if they were well, resident in the study area, planning to deliver their baby at the hospital, willing to participate and willing to know their HIV status. On the screening day, after giving written informed consent, eligible women were interviewed regarding sociodemographic characteristics and risk factors for helminth infection, malaria and HIV and were examined by a midwife. A blood sample was obtained for investigations including haemoglobin (Hb) estimation, examination for microfilariae (mf) and malaria parasites, syphilis and HIV serology.

Screened women were asked to return for enrolment within 1 month with a stool sample. They were excluded from enrolment if they had a Hb level <8 g/dl, clinically apparent severe liver disease, diarrhoea with blood in the stool, an abnormal pregnancy, a history of adverse reaction to anthelminthic drugs or had already participated in the study during an earlier pregnancy. Women were enrolled in the trial when they returned with a stool sample if they were in the second or third trimester and full eligibility was confirmed.

All women received routine antenatal care including haematinics and intermittent presumptive treatment for malaria using sulfadoxine/pyrimethamine. Women were treated for syphilis and provided with nevirapine for prevention of mother-to-child HIV transmission, if indicated. Women who were excluded from the study on grounds of severe anaemia were treated with albendazole and haematinics and referred for transfusion if required.

### 2.2 Haemoglobin estimation and definition of anaemia

Hb was estimated at the antenatal clinic using a colorimetric haemoglobinometer (DHT haemoglobin meter; Developing Health Technology, Barton Mills, UK) with same-day results. The same sample was then sent to the Medical Research Council/Uganda Virus Research Institute (MRC/UVRI) laboratories for analysis by a Coulter analyser (Beckman Coulter AC-T 5 diff CP; Beckman Coulter, Nyon, Switzerland). Quality control for the Coulter analyser was provided through the United Kingdom National External Quality Assessment Schemes, with consistently good results. Initial evaluation suggested that the haemoglobinometer results reliably matched the Coulter analyser results (intraclass correlation coefficient (ICC) = 0.81, 95% CI 0.74–0.88) and the immediately available haemoglobinometer results were used to determine enrolment. Evaluation of results to March 2005 showed less reliability (ICC = 0.54, 95% CI 0.51–0.58) and it was noted that 37 women had been enrolled with haemoglobinometer results >8 g/dl but Coulter analyser values below this cut-off; thereafter, Coulter analyser results were used for enrolment. Coulter analyser results have been used in this analysis.

In accordance with WHO criteria, anaemia was defined as Hb < 11.2 g/dl, i.e. 0.2 g/dl above the standard cut-off of 11 g/dl to allow for the altitude in Entebbe (1132 m a.s.l.) (http://www.sph.emory.edu/∼cdckms/hbadj2.html) (WHO, 1999a).

### 2.3 Parasitology

Examination of stool samples was performed as described previously (Bukusuba et al., 2004) using the Kato–Katz method (Katz et al., 1972) and charcoal culture for *Strongyloides* (Friend, 1996)*.* Two Kato–Katz slides were prepared from each sample, each examined within 30 min for hookworm or the following day for other parasites. Blood was examined for *Mansonella* by a modified Knott's method (Melrose et al., 2000). Intensity of infection was assessed by egg counts in stool and mf counts in blood. Intensities were categorised as follows: hookworm: light <1000 eggs per gram of stool (epg), moderate 1000–3999 epg, high ≥4000 epg (WHO, 1994); *Schistosoma mansoni*: light <100 epg, moderate 100–399 epg, high ≥400 epg (WHO, 1999b); *Trichuris trichiura*: light <1000 epg, moderate 1000–9999 epg, high ≥10 000 epg (WHO, 1999b). No standard categories are available for *Mansonella* intensity, therefore arbitrary categories were defined to obtain approximately equal numbers of participants in each category: light <30 mf/ml, moderate 30–99 mf/ml, high ≥100 mf/ml.

### 2.4 HIV serology

HIV serology was performed using a rapid test algorithm with same-day results. Testing kits, provided by the Ministry of Health, varied with availability. Most commonly, Determine (Abbott Laboratories, Abbott Japan Co. Ltd., Tokyo, Japan) was used for screening, with positive results confirmed by Unigold (Trinity Biotech plc, Bray, Ireland). Samples with differing results were referred for analysis by non-rapid ELISA tests or were examined using a ‘tie-breaker’ rapid test, usually Statpack (Chembio Diagnostic Systems, Medford, NY, USA). A proportion of samples, including all those with differing results by rapid test, were re-examined at the MRC/UVRI laboratories for quality control, with high agreement of the results.

### 2.5 Demographic information and potential risk factors for anaemia

**Figure 1:**
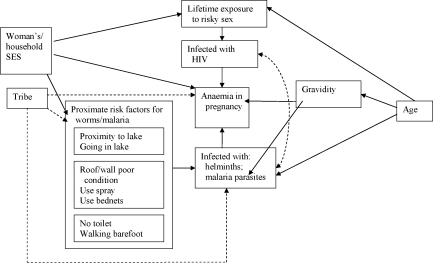
Hypothesised associations between helminth infections and anaemia, and measured potential confounding factors. Arrows indicate hypothesised associations, with direction indicating expected direction of effects. Dotted arrows indicate less certain effect. SES: socioeconomic status.

Socioeconomic data were summarised by developing two indices based on the variables that appeared to describe socioeconomic status most usefully. These were ‘woman's socioeconomic index’, comprised of education, personal income and occupation, and ‘household socioeconomic index’, comprised of building materials, number of rooms and items collectively owned. The relationship between potential confounding factors and anaemia and helminths, malaria and HIV were considered, and a diagram describing hypothesised relationships was developed (Figure 1). Age, socioeconomic status and tribe were considered to be possible confounders, with gravidity of potential importance, particularly for malaria, to which primigravidae are known to be particularly susceptible.

### 2.6 Data management and statistical analysis

Data were entered using Microsoft Access (Microsoft Corp., Redmond, WA, USA) and analysed using STATA version 8 (Stata Corp., College Station, TX, USA). The initial analysis was based on a binary variable for outcome (anaemic/not anaemic) and categorical variables for exposure, with binary variables for exposures to helminths, malaria and HIV infection (infected/not infected). Effects of helminth infection were then investigated in more detail by examining categories of infection intensity (none, light, moderate or heavy). For these analyses, cross-tabulations were made between selected risk factors and the presence of anaemia. Logistic regression was used to estimate unadjusted and adjusted odds ratios (OR). Likelihood ratio tests were used to determine *P*-values. Continuous variables for anaemia (Hb level) and infection intensity (egg or parasite count) were then used to examine effects of intensity in more detail among infected participants using linear regression. Adjustment was made for the same potential confounders in logistic and linear regression models.

## 3 Results

A total of 15 035 women registered at the antenatal clinic during the recruitment period, of whom 11 783 were assessed for inclusion in the study and 3163 were considered eligible and screened. The commonest reasons for ineligibility were residence outside the study area (6243), unwillingness to have an HIV test (1186), unwillingness to join the study (874) and enrolment during an earlier pregnancy (115). Of the 3163 screened, 2515 were enrolled; 8 of these were subsequently excluded because they had been enrolled during a previous pregnancy. Of the 648 women screened but not enrolled, the majority (596) failed to return for enrolment and only 15 brought a stool sample. Since intestinal helminth infection detected by stool analysis was a focus of interest, this analysis was confined to the 2507 women who were enrolled in the trial and for whom all or almost all relevant data were available.

### 3.1 Characteristics of the study women

Maternal age ranged from 14 years to 47 years (mean 23.6 years). The majority were Baganda (49.1%), the predominant tribe of the district. Most (83.8%) were married, with 13.4% single, 0.6% widows and 2.3% divorced or separated. Education varied from none (3.9%), to primary (50.5%), secondary (37.3%) and tertiary (8.4%) and most women were poor (85.1% with a personal income of less than £10 per month). Primigravidae comprised 27.7% of the women studied.

### 3.2 Prevalence of infections and anaemia

Complete data were obtained for Kato–Katz assays from 2498 women, for *Strongyloides* assays from 2485 women, for *Mansonella* from 2499 women and for malaria from 2459 women. The prevalence of hookworm was 44.5%, *Mansonella perstans* 21.3%, *S. mansoni* 18.3%, *Strongyloides stercoralis* 12.3%, *T. trichiura* 9.1%, *Ascaris lumbricoides* 2.3%, *Trichostrongylus* sp. 1.0%, *Hymenolepis nana* 0.2% and *Loa loa* <0.1%. Ova of *Fasciola hepatica* and *Dicrocoelium dendriticum* were found in samples from one and five women, respectively, but infection was not confirmed in follow-up samples and eggs probably originated from liver or offal in the women's diet; detection of ova of these two species was not considered to indicate infection. The prevalence of asymptomatic *Plasmodium falciparum* malaria parasitaemia was 10.9% and HIV infection 11.9%.

The prevalence of anaemia (Hb < 11.2 g/dl) was 39.7%. Six women were enrolled in error with Hb < 8 g/dl by both methods (Coulter analyser results 5.6–7.9 g/dl). These, as well as the 37 women enrolled prior to March 2005 with haemoglobinometer results above but Coulter analyser results below 8 g/dl, have been retained in this analysis.

### 3.3 Associations between characteristics of pregnant women and anaemia in pregnancy

Relationships between characteristics of the participating women and anaemia are shown in Table 1. The prevalence of anaemia declined with age. Anaemia was associated with tribe, with Basoga most likely to be anaemic. Anaemia showed no association with women's socioeconomic index, but low household socioeconomic status was associated with anaemia. Primigravidae were more likely to be anaemic than multigravidae.

**Table 1:** Associations between demographic characteristics of pregnant women and anaemia in pregnancy

Risk factor	Anaemia prevalence	Crude OR (95% CI)	*P*-value^a^ (trend)	Adjusted OR^b^ (95% CI)	*P*-value (trend)
	Proportion (%)				
Maternal age (years)					
14–19	285/630 (45.2)	1	0.025 (0.006)	1	0.060 (0.019)
20–24	366/946 (38.7)	0.76 (0.62–0.94)		0.76 (0.62–0.94)	
25–29	210/564 (37.2)	0.72 (0.57–0.91)		0.73 (0.57–0.94)	
30–34	92/252 (36.5)	0.70 (0.52–0.94)		0.71 (0.52–0.98)	
≥35	43/115 (37.4)	0.72 (0.48–1.09)		0.73 (0.47–1.13)	

Tribe					
Baganda	503/1231 (40.9)	1	0.020	1	0.020
Banyankole	82/234 (35.0)	0.78 (0.58–1.05)		0.78 (0.58–1.06)	
Batoro	31/102 (30.4)	0.63 (0.41–0.98)		0.61 (0.39–0.97)	
Basoga	54/106 (50.9)	1.50 (1.01–2.24)		1.57 (1.04–2.35)	
Luo	55/140 (39.3)	0.94 (0.65–1.34)		0.94 (0.64–1.38)	
Banyarwanda	64/142 (45.1)	1.19 (0.84–1.68)		1.14 (0.79–1.64)	
Other	206/551 (37.4)	0.86 (0.70–1.06)		0.87 (0.70–1.08)	

Mother's SES index^c^					
1 (low)	63/152 (41.4)	1	0.980 (0.488)	1	0.966 (0.894)
2	74/183 (40.4)	0.96 (0.62–1.49)		0.95 (0.61–1.48)	
3	339/849 (39.9)	0.94 (0.66–1.33)		0.90 (0.63–1.29)	
4	323/802 (40.3)	0.95 (0.67–1.35)		0.94 (0.65–1.35)	
5	69/182 (37.9)	0.86 (0.56–1.34)		0.87 (0.55–1.38)	
6 (high)	96/250 (38.4)	0.88 (0.58–1.33)		1.01 (0.66–1.54)	

Household SES index^d^					
1 (low)	64/147 (43.5)	1	0.049 (0.003)	1	0.127 (0.013)
2	88/217 (40.6)	0.88 (0.58–1.35)		0.88 (0.57–1.36)	
3	328/765 (42.9)	0.97 (0.68–1.39)		0.99 (0.68–1.43)	
4	274/710 (38.6)	0.82 (0.57–1.17)		0.83 (0.57–1.20)	
5	173/485 (35.7)	0.72 (0.49–1.05)		0.76 (0.51–1.12)	
6 (high)	43/134 (32.1)	0.61 (0.38–1.00)		0.62 (0.38–1.04)	

Gravidity (pregnancies)					
1	319/693 (46.0)	1	0.0002	1	0.014
2–4	519/1412 (36.8)	0.68 (0.57–0.82)		0.72 (0.58–0.90)	
≥5	157/400 (39.3)	0.76 (0.59–0.97)		0.81 (0.56–1.17)	

OR: odds ratio; SES: socioeconomic status.

a *P*-values are from the likelihood ratio test.

b Adjusted OR estimated from multivariate logistic regression models that included age, tribe, SES index and gravidity.

c Woman's SES is a score based on education, personal income and occupation. High scores indicate high status.

d Household SES is a score based on building materials, number of rooms and items owned. High scores indicate high status.

### 3.4 Relationship between infections and anaemia in pregnancy

Relationships between infections and anaemia are shown in Table 2. None of the helminths showed an association with anaemia when considered using categorical variables for the presence or absence of infection. Hookworm showed a weak positive association, but this was reduced in the adjusted model.

**Table 2:** Associations between infections (helminths, malaria and HIV) and anaemia in pregnancy

Infection	Anaemia prevalence	Crude OR (95% CI)	*P*-value^a^	Adjusted OR^b^ (95% CI)	*P*-value
	Proportion (%)				
Hookworm					
No	530/1386 (38.2)	1		1	
Yes	465/1112 (41.8)	1.16 (0.99–1.36)	0.070	1.07 (0.90–1.27)	0.431

*Mansonella*					
No	773/1968 (39.3)	1		1	
Yes	223/531 (42.0)	1.12 (0.92–1.36)	0.257	1.03 (0.84–1.27)	0.768

*Schistosoma mansoni*					
No	815/2040 (40.0)	1		1	
Yes	180/458 (39.3)	0.97 (0.79–1.20)	0.797	0.95 (0.76–1.18)	0.617

*Strongyloides*					
No	865/2179 (39.7)	1		1	
Yes	127/306 (41.5)	1.08 (0.85–1.37)	0.547	1.06 (0.82–1.36)	0.663

*Trichuris*					
No	904/2272 (39.8)	1		1	
Yes	91/226 (40.3)	1.02 (0.77–1.35)	0.889	0.95 (0.71–1.28)	0.738

Malaria parasites					
No	807/2191 (36.8)	1		1	
Yes	175/268 (65.3)	3.23 (2.47–4.21)	<0.001	3.22 (2.43–4.26)	<0.001

HIV status					
Negative	825/2208 (37.4)	1		1	
Positive	171/299 (57.2)	2.24 (1.75–2.86)	<0.001	2.46 (1.90–3.19)	<0.001

OR: odds ratio.

a *P*-values are from the likelihood ratio test.

b Adjusted OR estimated from multivariate logistic regression models that included age, tribe, socioeconomic indices and gravidity.

Malaria parasitaemia and HIV infection were strongly associated with anaemia; for malaria the effect was unchanged and for HIV it increased after adjusting for potential confounding factors. Malaria was also associated with HIV infection (OR 1.81, 95% CI 1.30–2.53; *P* = 0.001); the association between HIV and anaemia was reduced slightly, but not explained, when malaria was added to the model (adjusted OR (AOR), adjusted for age, tribe, socioeconomic index, gravidity and malaria: 2.27, 95% CI 1.74–2.97; *P* < 0.001). The likelihood of being anaemic was particularly high among women with both HIV infection and malaria compared with women with neither infection (AOR 6.50, 95% CI 3.27–12.95; *P* < 0.001). Comparing those with HIV only to those with neither gave an AOR of 2.30 (95% CI 1.73–3.05; *P* < 0.001) and comparing those with malaria only to those with neither gave an AOR of 3.09 (95% CI 2.28–4.20; *P* < 0.001).

Hookworm and *Mansonella* showed positive associations with malaria parasitaemia, and hookworm showed a negative association with HIV infection, but adjusting for these infections had minimal effect on the observed associations between helminths and anaemia (data not shown).

Attributable fractions for anaemia were 3.1% for hookworm, 12.3% for malaria and 10.2% for HIV infection.

### 3.5 Relationship between anaemia and infection intensity

Associations between infections and anaemia were further explored by examining infection intensity (Table 3). The prevalence of anaemia increased with each category of hookworm infection intensity. However, this trend was greatly weakened, showing no evidence of association after adjusting for age, tribe, socioeconomic index, gravidity, malaria and HIV. On the other hand, there was a small negative association between log_10_ hookworm egg count and Hb in hookworm-infected women (adjusted regression coefficient −0.23, 95% CI −0.38 to −0.09; *P* = 0.002), i.e. women with the highest hookworm intensity (approximately 10 000 epg) had, on average, a Hb level 0.69 g/dl lower than those with the lowest detectable egg counts (12 epg). Anaemia prevalence was higher in women with heavy *S. mansoni* infection than in those with lower intensity infection or no infection, but the number of such women was small and the effect was not statistically significant. Infections with *Trichuris* were light in all but six of the infected women, therefore associations between anaemia and moderate-to-heavy *Trichuris* infections could not be examined. There was no evidence of an association between Hb and egg count for women infected with *S. mansoni* or *Trichuris*.

**Table 3:** Association between intensity of helminth infections and anaemia in pregnancy

Helminth/categories of intensity	Anaemia prevalence		Crude OR (95% CI)	*P*-value^b^	Adjusted OR^c^ (95% CI)	*P*-value	Adjusted OR^d^ (95% CI)	*P*-value
	Proportion (%)	*P*-value^a^						
Hookworm								
Uninfected	530/1386 (38.2)	0.035	1	0.007	1	0.112	1	0.245
Light (<1000 epg)	382/942 (40.6)		1.10 (0.93–1.31)		1.09 (0.87–1.36)		1.03 (0.80–1.33)	
Moderate (1000–3999 epg)	59/127 (46.5)		1.40 (0.97–2.02)		1.61 (0.96–2.72)		1.74 (0.98–3.11)	
Heavy (≥4000 epg)	24/43 (55.8)		2.04 (1.11–3.77)		1.71 (0.75–3.92)		1.26 (0.49–3.20)	

*Mansonella*								
Uninfected	773/1968 (39.3)	0.637	1	0.445	1	0.490	1	0.262
Light (<30 mf/ml)	85/197 (43.1)		1.17 (0.87–1.58)		0.91 (0.62–1.33)		0.74 (0.47–1.16)	
Moderate (30–99 mf/ml)	50/116 (43.1)		1.17 (0.80–1.71)		1.02 (0.63–1.65)		1.00 (0.57–1.76)	
Heavy (≥100 mf/ml)	88/218 (40.4)		1.05 (0.79–1.39)		0.86 (0.60–1.24)		0.81 (0.53–1.25)	

*Schistosoma*								
Uninfected	815/2040 (40.0)	0.184	1	0.943	1	0.661	1	0.615
Light (<100 epg)	118/297 (39.7)		0.99 (0.77–1.27)		0.81 (0.59–1.12)		0.76 (0.52–1.11)	
Moderate (100–399 epg)	26/85 (30.6)		0.66 (0.41–1.06)		0.55 (0.29–1.05)		0.53 (0.24–1.19)	
Heavy (≥400 epg)	36/76 (47.4)		1.35 (0.85–2.14)		1.44 (0.79–2.62)		1.53 (0.78–2.98)	

OR: odds ratio; epg: eggs per gram of stool; mf: microfilariae.

a *P*-values are from the χ^2^ test for heterogeneity.

b *P*-values are from the χ^2^ test for trend.

c Adjusted for age, tribe, socioeconomic indices and gravidity, but not malaria or HIV.

d Adjusted for age, tribe, socioeconomic indices, gravidity, malaria and HIV.

There was a negative association between log_10_ malaria parasite count (parasites per 200 white blood cells) and Hb, but this was reduced in the adjusted model (crude regression coefficient −0.27, 95% CI −0.53 to −0.01, *P* = 0.039; adjusted regression coefficient −0.17, 95% CI −0.45 to 0.11, *P* = 0.206). Hb was not significantly associated with log_10_ CD4+ T-cell count among HIV-positive women (adjusted regression coefficient 0.51, 95% CI −0.17 to 1.19; *P* = 0.120).

### 3.6 Anaemia in women excluded from enrolment

Hb < 8 g/dl was an exclusion criterion. Among the 648 women who were screened but never enrolled, Hb < 8 g/dl was the reason given in 17 cases, but Coulter analyser Hb was <8 g/dl for a further 37 women excluded for other reasons. The prevalence of anaemia was 281/648 (43.4%) among those excluded compared with 996/2507 (39.7%) among those enrolled (*P* = 0.093), giving an overall prevalence of 40.5% among all women screened. Since stool results were only available for 15 of the excluded women, associations with intestinal helminths could not be analysed. Of the 54 women excluded and with Coulter Hb < 8 g/dl, two had stool results: both had hookworm infections (one light and one moderate intensity) and neither had schistosomiasis. A sensitivity analysis was performed assuming that all women excluded and with Coulter Hb < 8 g/dl had hookworm. As expected, a slightly stronger association was obtained, but this was again reduced after adjusting for confounding factors (crude OR 1.29, 95% CI 1.10–1.51, *P* = 0.001; AOR adjusted for age, tribe, socioeconomic indices and gravidity 1.19, 95% CI 1.00–1.41, *P* = 0.047).

Associations with malaria and HIV were similar in the excluded group to those in the enrolled group, with crude ORs of 3.73 (95% CI 2.23–6.23; *P* < 0.001) and 1.57 (95% CI 1.06–2.34; *P* = 0.026), respectively.

## 4 Discussion

This study suggests that, among pregnant women in Entebbe, Uganda, malaria and HIV are more important infectious causes of anaemia than helminths. No association was observed between mild-to-moderate anaemia and any species of helminth, and a weak association between anaemia and increasing intensity of hookworm infection was reduced after adjusting for confounding factors. Anaemia was slightly more common among women heavily infected with *S. mansoni*, but the number of such women was small.

In keeping with the exclusion of women with Hb < 8 g/dl, excluded women were more likely to be anaemic than enrolled women, but this had minimal impact on the overall estimate of prevalence of anaemia (40.5%). Given the recognised ability of hookworm to cause anaemia (Bondevik et al., 2000, Hotez et al., 2004, Shulman et al., 1996), we conducted a sensitivity analysis to estimate the effect of hookworm if all women with Hb < 8 g/dl and no stool result had hookworm. The result was in keeping with a possible effect of hookworm, but after adjusting for confounding factors the effect was small. Associations between anaemia and malaria and between anaemia and HIV infection were similar in enrolled and excluded women.

Our investigations for intestinal helminths used only one stool sample from each woman, meaning that a proportion of women with low-intensity infections will have been misclassified as uninfected and that estimates of intensity will have been imprecise (Hall, 1981, Utzinger et al., 2001). Recent comparable studies have the same limitation (Ajanga et al., 2006, Bondevik et al., 2000, Dreyfuss et al., 2000, Larocque et al., 2005). In our study this may, again, be important for hookworm, where there was a small increase in anaemia with infection intensity, but may not explain the lack of association for schistosomiasis where there was no suggestion of an effect of light-to-moderate infections. Our method of investigation for *Mansonella* and intensity does not suffer from this limitation: repeat examinations among 1971 women showed 96% agreement for the binary variable (infected/uninfected), with an ICC for mf/ml of 0.87 (95% CI 0.85–0.88) (unpublished data).

Not all pregnant women in Entebbe attend the district hospital antenatal clinic, but a community survey undertaken in the study area showed an increase in the proportion choosing this clinic during the recruitment period, to approximately 80%, and most of the personal and socioeconomic characteristics of women choosing, or not choosing, to attend the district hospital clinic were similar (unpublished data). Thus, our results are likely to be reasonably representative of pregnant women in this area.

Recent studies from different settings give similar results in relation to the effects of hookworm and *S. mansoni* on anaemia in pregnancy. In Peru (where hookworm and *Trichuris* are predominant), in Tanzania (*S. mansoni* and hookworm), in the Democratic Republic of Congo (*Ascaris* and hookworm) and in Java (*Trichuris* and hookworm), no association was observed between anaemia and infection with any single species (Ajanga et al., 2006, Kalenga et al., 2003, Larocque et al., 2005, Nurdia et al., 2001). In Peru there was an association between anaemia and higher hookworm intensities, and in Java there was a negative association between serum ferritin and hookworm, suggesting an effect of hookworm on iron status but not anaemia; in Tanzania, in an area of higher *S. mansoni* prevalence and intensity, a strong association between anaemia and heavy *S. mansoni* infection was observed. Infection intensity thus appears to be important, with light-to-moderate hookworm or *S. mansoni* infections having relatively weak effects on Hb levels. However, a second important factor is the underlying nutritional status of the women. In two studies in Nepal, among populations perhaps poorer and less well nourished than ours (as indicated by items owned, anthropometry and vitamin A status (Dreyfuss et al., 2000) and with a traditionally vegetarian diet (Bondevik et al., 2000)), anaemia showed a significant association with hookworm infection (Bondevik et al., 2000, Dreyfuss et al., 2000). The effects of hookworm infection are partially mediated by iron deficiency (Bondevik et al., 2000, Olsen et al., 1998) and in the trial conducted in Sierra Leone iron-folate supplements had a greater benefit for anaemia in pregnancy than treatment with albendazole (Torlesse and Hodges, 2001).

We found no suggestion of an association between anaemia and any other helminth species that was common in our environment (*Mansonella*, *Trichuris* or *Strongyloides*). This again is in agreement with other studies (Dreyfuss et al., 2000, Larocque et al., 2005, Nurdia et al., 2001). Larocque et al. (2005) noted a stronger effect of moderate-to-heavy hookworm infection when combined with moderate-to-heavy *Trichuris* infection. Although there was broad overlap between the confidence intervals for these effects in their analysis, such an effect is plausible, given evidence in children that heavy *Trichuris* infections (>10 000 epg) can be associated with anaemia (Ramdath et al., 1995). In our study, as in that reported by Nurdia et al. (2001), no such heavy *Trichuris* infections were observed.

The strong effects of malaria and HIV contrast with the weak effect of hookworm and the lack of effects of other helminths in this study.

Of note, women were screened for this study when they were apparently healthy, thus both malaria and HIV infection were largely asymptomatic. The effects of these infections are not mediated by iron deficiency and may override any benefit of good nutrition. The importance of malaria as a cause of anaemia in pregnancy is well established (Shulman and Dorman, 2003). HIV infection is also a recognised cause of anaemia (Belperio and Rhew, 2004), and anaemia may be one mechanism by which it causes adverse birth outcomes (Dairo et al., 2005, McIntyre, 2003).

There are compelling reasons for preventing and treating malaria and HIV during pregnancy (Shulman and Dorman, 2003, ter Kuile et al., 2004). Our results highlight that anaemia is among them. On the other hand, our results as well as recent literature suggest that associations between helminth infections and anaemia in pregnancy are weaker, with regional variations that may be based on nutrition and intensity of helminth infection. These findings are relevant when estimating the relative disease burden of helminths and other infections and the relative value of possible interventions in pregnancy. Globally, the majority of helminth infections are of low intensity so, in some settings, the benefit of routine deworming during pregnancy in relation to anaemia may be modest. The effects of deworming during pregnancy on other parameters, including birth outcome, birth weight and long-term effects on health in infancy and childhood, also need to be considered (Christian et al., 2004). The forthcoming results of our ongoing trial of deworming in pregnancy are expected to contribute further to this debate.

## Authors’ contributions

AME designed the study; JN and CA carried out interviews and recruited participants; MO and HM carried out clinical assessments; NO and DK carried out laboratory assessments; LM, PW and LAM analysed and interpreted the data; LM, PW, LAM and AME drafted the manuscript. All authors reviewed and approved the final manuscript. LM and AME are guarantors of the paper.

## Funding

Wellcome Trust Career Post fellowship held by Dr Elliott, grant number 064693; PMTCT programme, Ministry of Health, Uganda.

## Conflicts of interest

None declared.

## Ethical approval

The Science and Ethics Committee, Uganda Virus Research Institute, the Uganda National Council for Science & Technology, and the London School of Hygiene & Tropical Medicine.

## References

[bib1] Ajanga A., Lwambo N.J., Blair L., Nyandindi U., Fenwick A., Brooker S. (2006). *Schistosoma mansoni* in pregnancy and associations with anaemia in northwest Tanzania. Trans. R. Soc. Trop. Med. Hyg..

[bib2] Allen H.E., Crompton D.W.T., de Silva N., LoVerde P.T., Olds G.R. (2002). New policies for using anthelmintics in high risk groups. Trends Parasitol..

[bib3] Belperio P.S., Rhew D.C. (2004). Prevalence and outcomes of anemia in individuals with human immunodeficiency virus: a systematic review of the literature. Am. J. Med..

[bib4] Bondevik G.T., Eskeland B., Ulvik R.J., Ulstein M., Lie R.T., Schneede J., Kvale G. (2000). Anaemia in pregnancy: possible causes and risk factors in Nepali women. Eur. J. Clin. Nutr..

[bib5] Bukusuba J.W., Hughes P., Kizza M., Muhangi L., Muwanga M., Whitworth J.A., Elliott A.M. (2004). Screening for intestinal helminth infection in a semi-urban cohort of pregnant women in Uganda. Trop. Doct..

[bib6] Christian P., Khatry S., West K.P. (2004). Antenatal anthelmintic treatment, birthweight, and infant survival in rural Nepal. Lancet.

[bib7] Dairo M.D., Lawoyin T.O., Onadeko M.O., Asekun-Olarinmoye E.O., Adeniji A.O. (2005). HIV as an additional risk factors for anaemia in pregnancy: evidence from primary care level in Ibadan, Southwestern Nigeria. Afr. J. Med. Med. Sci..

[bib8] Dreyfuss M.L., Stoltzfus R.J., Shrestha J.B., Pradhan E.K., LeClerq S.C., Khatry E.K., Shrestha S.R., Katz J., Albonico M., West K.P. (2000). Hookworms, malaria and vitamin A deficiency contribute to anemia and iron deficiency among pregnant women in the plains of Nepal. J. Nutr..

[bib9] Elliott A.M., Kizza M., Quigley M.A., Ndibazza J., Nampijja M., Muhangi L., Morison L., Namujju P.B., Muwanga M., Kabatereine N., Whitworth J.A.G. (2007). The impact of helminths on the response to immunisation and on the incidence of infection and disease in childhood in Uganda: design of a randomised, double-blind, placebo-controlled, factorial trial of de-worming interventions delivered in pregnancy and early childhood. Clin. Trials.

[bib10] Friend, J., 1996. Helminths, in: Collee, J.G., Fraser, A., Marmion, B., Simmons, A. (Eds), Mackie & McCartney, Practical Medical Microbiology. Churchill Livingstone, Edinburgh, p. 757.

[bib11] Hall A. (1981). Quantitative variability of nematode egg counts in faeces: a study among rural Kenyans. Trans. R. Soc. Trop. Med. Hyg..

[bib12] Hotez P.J., Brooker S., Bethony J.M., Bottazzi M.E., Loukas A., Xiao S. (2004). Hookworm infection. N. Engl. J. Med..

[bib13] Kalenga M.K., Nyembo M.K., Nshimba M., Foidart J.M. (2003). Anemia prevalence in pregnant and breast-feeding women in Lubumbashi (Democratic Republic of the Congo). Impact of malaria and intestinal helminthiasis. J. Gynecol. Obstet. Biol. Reprod. (Paris).

[bib14] Katz N., Chaves A., Pellegrino N. (1972). A simple device for quantitative stool thick-smear technique in *Schistosomiasis mansoni*. Rev. Inst. Med. Trop. Sao Paulo.

[bib15] Larocque R., Casapia M., Gotuzzo E., Gvorkos T.W. (2005). Relationship between intensity of soil-transmitted helminth infections and anemia during pregnancy. Am. J. Trop. Med. Hyg..

[bib16] McIntyre J. (2003). Mothers infected with HIV. Br. Med. Bull..

[bib17] Melrose W.D., Turner P.F., Pisters P., Turner B. (2000). An improved Knott's concentration test for the detection of microfilariae. Trans. R. Soc. Trop. Med. Hyg..

[bib18] Nurdia D.S., Sumarni S., Suyoko, Hakim M., Winkvist A. (2001). Impact of intestinal helminth infection on anemia and iron status during pregnancy: a community based study in Indonesia. Southeast Asian J. Trop. Med. Public Health.

[bib19] Olsen A., Magnussen P., Ouma J.H., Andreasson J., Friis H. (1998). The contribution of hookworm and other parasitic infections to haemoglobin and iron status among children and adults in western Kenya. Trans. R. Soc. Trop. Med. Hyg..

[bib20] Ramdath D.D., Simeon D.T., Wong M.S., Grantham-MacGregor S.M. (1995). Iron status of schoolchildren with varying intensities of *Trichuris trichiura* infection. Parasitology.

[bib21] Shulman C.E., Dorman E.K. (2003). Importance and prevention of malaria in pregnancy. Trans. R. Soc. Trop. Med. Hyg..

[bib22] Shulman C.E., Graham W.J., Jilo H., Lowe B.S., New L., Obiero J., Snow R.W., Marsh K. (1996). Malaria is an important cause of anaemia in primigravidae: evidence from a district hospital in coastal Kenya. Trans. R. Soc. Trop. Med. Hyg..

[bib23] ter Kuile F.O., Parise M.E., Verhoeff F.H., Udhayakumar V., Newman R.D., van Eijk A.M., Rogerson S.J., Steketee R.W. (2004). The burden of co-infection with human immunodeficiency virus type 1 and malaria in pregnant women in sub-saharan Africa. Am. J. Trop. Med. Hyg..

[bib24] Torlesse H., Hodges M. (2000). Anthelminthic treatment and haemoglobin concentrations during pregnancy. Lancet.

[bib25] Torlesse H., Hodges M. (2001). Albendazole therapy and reduced decline in haemoglobin concentration during pregnancy (Sierra Leone). Trans. R. Soc. Trop. Med. Hyg..

[bib26] Utzinger J., Booth M., N’Goran E.K., Muller I., Tanner M., Lengeler C. (2001). Relative contribution of day-to-day and intra-specimen variation in faecal egg counts of *Schistosoma mansoni* before and after treatment with praziquantel. Parasitology.

[bib27] WHO, 1991. Maternal Health and Safe Motherhood Programme. Prevention and Management of Severe Anaemia in Pregnancy. Report of a Technical Working Group, 20–22 May 1991, Geneva, Switzerland. World Health Organization, Geneva, WHO/FHP/MSM/93.5.

[bib28] WHO, 1994. Report of the WHO Informal Consultation on Hookworm Infection and Anaemia in Girls and Women, 5–7 December 1994, Geneva, Switzerland. World Health Organization, Geneva, WHO/CTD/SIP/96.1.

[bib29] WHO, 1999a. Report of the UNICEF/WHO Regional Consultation. Prevention and Control of Iron Deficiency Anaemia in Women and Children, 3–5 February 1999, Geneva, Switzerland. http://www.euro.who.int/Document/E73102.pdf [accessed 22 March 2007].

[bib30] WHO (1999).

[bib31] WHO, 2002. Report of the WHO Informal Consultation on the Use of Praziquantel During Pregnancy/Lactation and Albendazole/Mebendazole in Children under 24 months, 8–9 April 2002, Geneva, Switzerland. World Health Organization, Geneva, WHO/CDS/CPE/PVC/2002.4.

